# Do patients with recurrent episodes of campylobacteriosis differ from those with a single disease event?

**DOI:** 10.1186/1471-2458-11-32

**Published:** 2011-01-12

**Authors:** Julie Arsenault, André Ravel, Pascal Michel, Olaf Berke, Pierre Gosselin

**Affiliations:** 1 Faculty of Veterinary Medicine, University of Montreal, 3200 Sicotte, Saint-Hyacinthe, Quebec, J2S 7C6, Canada; 2 Laboratory for Foodborne Zoonoses, Public Health Agency of Canada, 3200 Sicotte, Saint-Hyacinthe, Quebec, J2S 7C6, Canada; 3 Groupe de recherche en épidémiologie des zoonoses et santé publique, Université de Montréal, 3200 Sicotte, Saint-Hyacinthe, Quebec, J2S 7C6, Canada; 4 Department of Population Medicine, University of Guelph, Guelph, Ontario N1G2W1, Canada; 5 Institut national de santé publique du Québec (INSPQ), 945 Avenue Wolfe, Quebec City, Quebec, G1V 5B3, Canada; Centre hospitalier universitaire de Québec (CHUQ), 2705 boulevard Laurier, Sainte-Foy, Quebec, G1V 4G2, Canada

## Abstract

**Background:**

Although *Campylobacter *is the leading cause of reported bacterial gastro-enteritis in industrialized countries, little is known on its recurrence. The objective of this study is to describe the risk and the patient characteristics of recurrent episodes of human campylobacteriosis reported in Quebec.

**Methods:**

Laboratory-confirmed cases of campylobacteriosis reported in the province of Quebec, Canada, through ongoing surveillance between 1996 and 2006 were analyzed. The risk of having a recurrent episode of campylobacteriosis was described using life table estimates. Logistic regression was used to assess if gender, age and patient residential location were associated with an increased risk of recurrence.

**Results:**

Compared to the baseline risk, the risk for a recurrent disease event was higher for a period of four years and followed a decreasing trend. This increased risk of a recurrent event was similar across gender, but higher for people from rural areas and lower for children under four years of age.

**Conclusions:**

These results may suggest the absence of durable immunity or clinical resilience following a first episode of campylobacteriosis and periodical re-exposure, at least among cases reported through the surveillance system.

## Background

In Canada, infection by *Campylobacter *spp. is the leading cause of bacterial gastro-enteritis, with an average of 39 cases per 100,000 people reported annually over the last decade [1]. However, given the mild clinical expression of most *Campylobacter *infections, this reflects an underestimation of the true burden in the population, as only a fraction of people affected consult their physician and have stool samples submitted for culture [2].

In Canada as in many other countries, human campylobacteriosis is a reportable disease. Regional public health authorities are notified of confirmed cases and selected information is then gathered into surveillance databases. These databases can then be used in epidemiological investigations as sources of information for evaluating individual or environmental risk factors [3,4]. It was recently proposed that acquired immunity to the infection could bias results from risk factor analysis by reducing the risk of developing the disease in areas of high exposure [5].

Following an infection with *Campylobacter*, most people develop a humoral immune response. Circulating antibodies are detectable six to seven days after the onset of the disease [6]. The antibody peak occurs within 7 days to 4 weeks, depending on the specific serum immunoglobulin, and then declines over several months [6]. Although the development of antibodies following a *Campylobacter *infection is well recognized, their protective role is poorly understood, especially in populations living in developed countries [6].

One of the potential avenues of exploring the possible protective effect of immunity is through studying recurrence. Immunity following a first episode of campylobacteriosis would be associated with a reduced risk for subsequent events, which might also vary in time and according to patient characteristics. Characterization of the risk of recurrent episodes may also bring valuable insight to the question of whether or not to include patients with multiple reports of the disease in risk factor analyses. The objective of this study is therefore to describe the risk of having a recurrent episode of campylobacteriosis in relation to patient characteristics.

## Methods

This study was designed as a retrospective analysis of human campylobacteriosis cases reported through ongoing surveillance in the province of Quebec, Canada, between January 1, 1996 and December 31, 2006. Cases from non-organized territories, incompletely enumerated First Nations reserves, and areas located north of the 55th parallel were excluded.

### Case data

Following approval of the project by the research ethics board of the Montreal Health and Social Services Agency, all laboratory-confirmed cases of campylobacteriosis were retrieved from each the health region's reportable disease database. Case information extracted includes a de-identified record number, age, gender, *Campylobacter *species isolated, various dates related to onset and report, place of residence, and outcome. Cases were geocoded at the municipality level (using 2006 boundaries). Patients were then classified as living in an urban region if their municipality of residence was entirely within an urban area, as rural if entirely outside, or as semi-urban if otherwise. An urban area was defined according to Statistics Canada as an area with a population of at least 1,000 people and no fewer than 400 persons per km^2 ^(Statistics Canada, 2003).

### Population data

Population census data by age, gender, and census subdivisions (CSD) were obtained from Statistics Canada for the census years of 1996, 2001, and 2006. A weighted average annual population for each CSD was calculated over the three census years using 0.25, 0.5, and 0.25 weights, respectively. A weighted average annual population by urbanicity was also calculated using a similar method.

### Date of onset

For each reported case, the date of onset was retrieved directly from the database when available. If this date was missing, an estimation was performed using a "proxy date" based on the first of the following dates with a non-missing value: date of sample collection, date of physician notification, date of laboratory notification, date of reception of physician report by authorities, and/or date of reception of laboratory report by authorities. When date of onset had to be estimated, the median time period (in days, by health region) between the onset and proxy date was subtracted from a case's specific proxy date and used as the estimated date of onset.

### Recurrent episodes

Reported cases of recurrent episodes of campylobacteriosis were identified using unique de-identified record numbers. Episodes of campylobacteriosis occurring within 90 days of the date of onset of a prior episode were excluded sincethese cases were diagnosed within the recognized reported interval for duration of bacterial excretion [7,8] and in consideration of the 90 day cut-off time used by health officers for multiple entry detection and removal during the validation process of reportable disease databases [9].

### Descriptive analyses

Descriptive statistics were used to investigate the frequency distribution of patients according to the number of episodes of campylobacteriosis reported, and the distribution of the time lag between two consecutive episodes. For patients with multiple episodes, the association between *Campylobacter *species (*jejuni*, *coli*, etc.) isolated in first and second episodes was tested using the exact McNemar test and performed in SAS 9.2.

The baseline incidence of campylobacteriosis was estimated by taking all first episode cases and dividing them by the average population. This estimation was performed for all combined cases and then stratified by gender, age, and urbanicity. Age of patients was classified as 0-4 yrs, 5-14 yrs, 15-44 yrs, 45-64 yrs, and >65 yrs. A confidence interval (95%) for the average baseline incidence of campylobacteriosis was estimated under the normal distribution assumption, and results were then converted to an annual rate per 100,000 people.

### Survival analyses

The annual risk of having a recurrent episode of campylobacteriosis was estimated among reported cases using a survival analysis. These cases were then stratified by gender, age, and urbanicity and a survival analysis was performed again on each category. Survival function was inferred from life-table estimates in SAS 9.1. Time of entry of each patient into the analysis was set as the date of onset of the first episode plus 90 days. However, for the analysis by age group, time of entry was set as the day at which the patient reached the lower bound of the age group studied plus 90 days. For the analysis by urbanicity, the category was determined by the type of region noted upon first episode, since information on changes in residency regions was not complete. Time-to-event was estimated as the interval of time between the time of entry and a second episode. Right censoring was used for patients with no other reported episodes, with time-to-censoring calculated as the time between entry in the analysis and the last day of the study. For the analysis by age group, right censoring was also used when the age of the patient reached the upper bound of the age group studied. The risk of campylobacteriosis with a 95% confidence interval was estimated at the mid-point of 6-month intervals, and the risk was converted to an annual rate per 100,000 people. However, for the analyses by gender, age, or urbanicity, the studied time period was restricted to the 5 years following the first episode, with the risk calculated at the mid-point of this 5-year interval. Due to the low number of cases in these stratum-specific analyses, the use of 6-month intervals would have led to very large confidence intervals.

### Logistic regression

Logistic regression was used to evaluate patient characteristics in relation to the occurrence of reported episodes of campylobacteriosis. Three models were developed, one for each patient characteristic studied (i.e., gender, age group, and urbanicity). Given that we did not collect direct information from non-cases, a simulated individual database was created for each model, taking into consideration information from the population-at-risk over a 5 year period. The outcome was the report of an episode of campylobacteriosis (yes, no) and the following explanatory variables were included: previous episode of campylobacteriosis (yes, no), characteristics of patient (as categories), and their interaction. The database was created in two steps. First, the number of records for people without a previous reported episode of campylobacteriosis was estimated separately for gender, age group, or urbanicity as the average population at risk in the study area based on census information. Among these, a number of records corresponding to the average number of reported cases (first episode) for a 5 year period were set as having had a positive outcome. For example, in the model including gender, 3,519,216 males were included in the analysis as having had no previous reported episode of campylobacteriosis; among them, 7155 were set as having had a positive outcome (i.e. 15,742 reported male cases for the study period/11 years of the study period*5 years) and the remaining 3,512,061 males were set as having had a negative outcome (see Table 1). In the second step, records for people with a previous episode were added to the database and set as the effective population at risk for each category of patient characteristics estimated from the survival analysis for the 5 years following a reported episode of campylobacteriosis (see Table 1). Outcome was then set as positive for the number of patients corresponding to the number of second episodes of campylobacteriosis reported (for example, 12,485 males were included as having had a first episode, among which 163 had a positive outcome). Most patients with two episodes of campylobacteriosis were included twice in the analysis. A hierarchical backward procedure was used for model selection using P ≥ 0.05 based on the likelihood ratio test (LRT) as criterion for removal (performed in SAS 9.2). The odds ratio (OR) was used to present results.

**Table 1:** Incidence rates according to patient characteristics

	First episode (11 years of follow-up)			Second episode (5 years of follow-up)			Ratio in rates^c^
			
Charac-teristics	Average population at risk per year	Cases^a^	Episode per year per 100,000 (95% C.L)	Effective population at risk^b^	Cases^a^	Episode per year per 100,000 (95% C.L)	
*Gender*							
Male	3,519,216	15,742	40.7 (40.0, 41.3)	12,485	163	262.8 (222.5, 303.2)	6.5
Female	3,687,208	13,142	32.4 (31.9, 33.0)	10,540	121	230.9 (189.8, 272.1)	7.1
*Age category (yrs)*^d^							
≤4	389,854	2,406	56.1 (53.9, 58.4)	1,272	16	252.9 (129.0, 376.8)	4.5
5-14	890,700	2,774	28.3 (27.3, 29.4)	2,406	22	183.6 (106.9, 260.3)	6.5
15-44	3,097,425	15,351	45.1 (44.3, 45.8)	8,252	176	430.7 (367.1, 494.3)	9.5
45-64	1,872,161	5,262	25.6 (24.9, 26.3)	3,244	40	247.8 (171.0, 324.6)	9.7
≥65	955,915	3,032	28.8 (27.8, 29.9)	1,944	26	269.0 (165.6, 372.4)	9.3
*Urbanicity*^e^							
Rural	893,415	4,209	42.8 (41.5, 44.1)	3,247	62	385.6 (289.6, 481.6)	9.0
Semi-urban	3,438,014	15,876	42.0 (41.3, 42.6)	12,780	170	267.8 (227.6, 308.1)	6.4
Urban	2,875,304	8,632	27.3 (26.7, 27.9)	6,889	51	148.6 (107.8, 189.4)	5.4
*Total*	7,206,733	28,905	36.5 (36.0, 36.9)	23,041	284	248.0 (219.2, 276.9)	6.8

Incidence rate of reported cases of campylobacteriosis with 95% confidence limits for first and consecutive episodes according to patient characteristics, Quebec, 1996-2006

^a ^Total number of cases observed during the follow-up period.

^b ^The effective population at risk (n_i_') is the total number of individuals followed in the 5-year period (n_i_) minus half the number of individuals censored (w_i_) (n_i_' = n_i_-w_i_/2).

^c ^Estimated incidence rate for second episode vs. first episode.

^d ^For the first episode, the age category is the age at the time of the episode (case) and census data for the age group (population). For the second episode, patients were included in the analysis only for the period during which they were in the age category and if they had had an episode in the previous 5 years.

^e ^Region of residence at time of first episode.

## Results

A total of 29,407 episodes of laboratory-confirmed cases of campylobacteriosis were recorded in the surveillance database between 1996 and 2006, inclusively. From these, 158 were excluded because a previous episode was recorded within 90 days of the date of onset. The remaining 29,249 episodes were linked to 28,905 patients for an average of 1.01 episodes per patient. Mortality related to campylobacteriosis was recorded for 6 patients.

**Figure 1:**
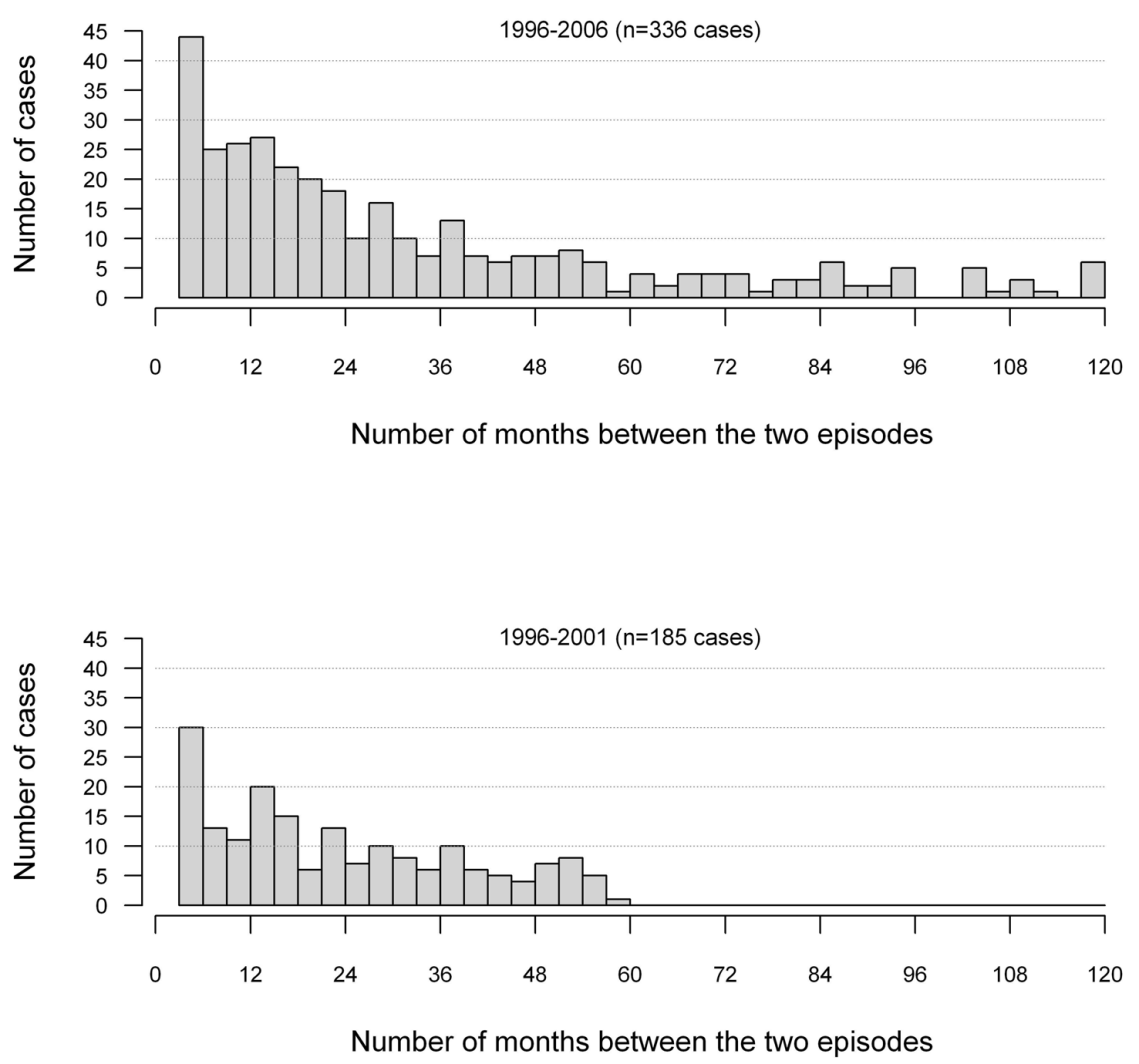
**Interval of time between reported episodes**. Interval of time between a first and a second episode of campylobacteriosis in laboratory-confirmed reported cases in Quebec, 1996-2006 (episodes occurring in the first 90 days following the first episode were not considered). Top: all cases. Bottom: cases reported between 1996 and 2001 and excluding episodes recurring after 5 years or more.

In assessing the completeness of information for the estimation of the date of onset, the percentages of records with valid information were: 72% for a date of sample collection, 15% for date of physician notification, 16% for date of reception of physician report, 96% for date of laboratory notification, 99% for date of reception of laboratory report, and 38% had a valid date of onset. Consequently, the onset date was mostly estimated based on the actual reported date of onset (38%) or on the date of laboratory notification (61%). The various intervals of time used for the estimation showed important variations among health regions, as presented in Additional file 1: Proxy dates for estimating date of onset. For the 28,905 patients, 328 (1.1%) had 2 reported episodes, 8 (0.03%) had three reported episodes, and none had more than 3 episodes. The distribution of the lag time between the first and second episode is presented in Figure 1. Among the 336 patients with recurrent episodes, 90% resided in the same municipality and 93% in the same region (rural, semi-urban, or urban) at the time of their first two reported episodes. The percentage of patients who had moved from an urban region to another region type between their two episodes was similar to the percentage that had moved from a rural region to another one (9.9% versus 8.1%).

No statistically significant association was observed between the *Campylobacter *species isolated in the first and second episode (P = 1.00, McNemar exact test). This analysis was restricted to patients with only *Campylobacter jejuni *or *Campylobacter coli *isolated in their first or second episode (n = 210), considering the low number of reported cases for other species (Table 2). It is worth noting that among cases with *Campylobacter coli *isolated in the first episode, 36.8% also had *Campylobacter coli *isolated in the second episode, although *Campylobacter coli *was noted in only 5% of episodes in the entire dataset (considering only episodes with a single species isolated). Further exploration of the data reveals that among these 210 isolates, the proportion of *Campylobacter coli *isolates was similar according to the level of urbanicity, estimated at 5.8% in semi-urban areas, 11.5% in rural areas and 11.3% in urban areas (P = 0.25, Chi-square test).

**Table 2:** *Campylobacter *species isolated

First episode	Second episode (within a 5-year interval)		
	
	Species	Number	%
*jejuni*	*jejuni*	172	92.5
	*coli*	11	5.9
	*jejuni *and *coli*	1	0.5
	*hyointestinalis*	1	0.5
	*fetus*	1	0.5
	*Total*	*186*	*100.0*
*coli*	*jejuni*	12	63.2
	*coli*	7	36.8
	*jejuni *and *coli*	0	0.0
	*Total*	*19*	*100.0*
*jejuni *and *coli*	*jejuni*	3	60.0
	*coli*	2	40.0
	*jejuni *and *coli*	0	0.0
	*Total*	*5*	*100.0*

*Campylobacter *species isolated during the first two consecutive episodes of campylobacteriosis among laboratory-confirmed cases reported in Quebec, 1996-2006 (n = 210 patients with recurrent episodes)

### Description of the risk of recurrent episode

**Figure 2:**
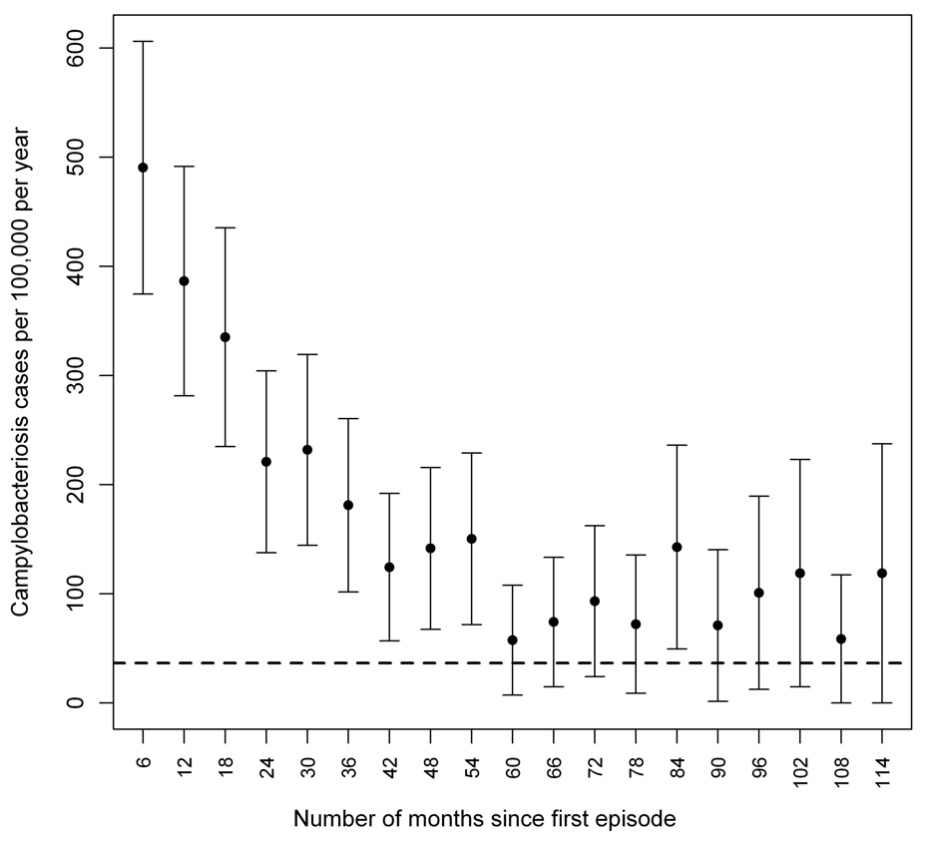
**Risk of campylobacteriosis after a first episode**. Risks of campylobacteriosis (with 95% confidence limits) after a first episode among laboratory-confirmed reported cases in Quebec, 1996-2006. Risks were calculated at the mid-interval of 6-month periods following the first episode onset +90 days (dotted line: average incidence rate of first episode in the population).

The overall incidence rate of recurrent campylobacteriosis decreased from 490 per 100,000 people per year within the first 6 months following an episode, to 150 per 100,000 people per year by 4 years after the first episode (Figure 2). After 5 to 9 years, the incidence rate was not statistically different from the incidence observed in the population for a first episode. An increased risk was also observed among all age, gender, and urbanicity groups with a risk for a second episode 5.4 to 9.7 times higher than the risk estimated for a first episode (Table 1).

According to the logistic regression model for gender, no statistically significant interaction was observed (P = 0.42, LRT) between gender and a previous episode of campylobacteriosis within the ensuing 5 years, suggesting that a previous episode increased the risk of campylobacteriosis by the same magnitude for both genders. This interaction was thus removed from the model. The estimates for gender (OR = 1.3 for male versus female) and a previous episode (OR: 6.8) were statistically significant (P < 0.001, LRT). For the logistic regression model including age, the interaction between age and a previous episode was statistically significant (P = 0.03, LRT). According to post-hoc pairwise comparisons, the increase in the risk of campylobacteriosis following a first episode was lower in the 0-4 age group compared to the other age groups (OR = 2.1 to 2.2, P > 0.02). Finally, according to the logistic regression model including urbanicity, a statistically significant interaction (P = 0.02, LRT) was observed between the type of region and a previous episode. Post-hoc pairwise comparisons revealed that the impact of having had a previous episode of campylobacteriosis on the risk of a subsequent episode was greater in rural areas compared to semi-urban (OR = 1.4, P = 0.02) or urban (OR = 1.7, P = 0.008) areas.

## Discussion

According to our results, the risk of a recurrent episode of campylobacteriosis was 248 for every 100,000 patients per year on average for the first 5 years following a first episode. This means that approximately 1.2% of patients with a reported episode of campylobacteriosis suffer from another episode within the next five years. Although this percentage might seem low, it represents a much higher risk than the one observed in the general population.

The relatively high rate of recurrence in campylobacteriosis cases may suggest that people in Quebec, like in other regions of industrialized countries, might be exposed to *Campylobacter *at lower levels than needed to develop an effective immunity. This would be in agreement with other studies which conclude that the development of immunity against *Campylobacter *is most likely limited to developing countries, where individuals are most likely exposed to a highly contaminated environment, characterized for example by a poor quality water supply or by the presence of poultry in the house [5,8,10,11]. Partial immunity in developing countries is supported by children excreting the bacteria for shorter duration and by disproportionately higher risk in travellers [12]. In industrialized countries, the exposure to *Campylobacter *is considered much lower, which translates into lower incidence rates of campylobacteriosis in children and a lower percentage of carriers [11,13,14]. One of the few documented instances of acquired immunity to *Campylobacter *infection in industrialized countries is with people professionally exposed, such as poultry abattoir workers [15,16]. This fraction of the population, although likely present among the cases reported in the province of Quebec, is probably too small to have had an impact in our study. Another study conducted in Scotland found some evidence of age-acquired immunity to common serotypes [17], whereas immunity to common genotypes was proposed as an explanation for reduction in incidence of campylobacteriosis in some countries [18].

Our data did not show evidence of the development of *Campylobacter *species-specific immunity, since people with recurrent episodes were as likely to have been infected twice by the same *Campylobacter *species. However, *Campylobacter *strains for the same species are reported to present a high genetic diversity [19]. Thus, immunity is expected to develop for strains sharing similar antigenic properties, which could not be evaluated in this study. This analysis could have been biased by the exclusion of some cases involving other or multiple species; however, this exclusion amounted to only 1% of cases. It should be remembered that reported cases represent only a small fraction of all cases occurring in the population. It would be interesting to evaluate if patients with more severe clinical presentations are similar in terms of immune response and risk of recurrence when compared to milder cases.

In addition to the lack of evidence of an acquired immunity, a prior episode seems to increase the risk of reporting a second episode of campylobacteriosis. Campylobacteriosis might cause long-term perturbations of intestinal physiology leading to an increased susceptibility to enteric diseases in general [20]. Long-term patho-physiological consequences of *Campylobacter *infections are also supported by a Danish study that noted an increased mortality following a reported episode of campylobacteriosis for the year following the episode, even after adjustment for comorbidity [21]. Unfortunately, no information was available on the significance of relapses or recurrent episodes in the explanation of this excess risk.

It is also possible that the higher risk is driven by some sub-groups of the population with diseases that predispose them to a high risk of recurrence. In the literature, there are reported cases of patients with hypogammaglobulinemia who experienced recurrent episodes of campylobacteriosis [22]; however, recurrent episodes were closer in time than what we observed and a predisposing disease such as this is rare. Other diseases were reported as risk factors for campylobacteriosis, including AIDS, chronic intestinal diseases, diabetes, liver disease and metastatic cancers [21,23-25]. The use of antacid or antibiotics is also associated with a higher risk of campylobacteriosis [26-28]. This can also be the case for people using corticosteroids. These drugs are commonly prescribed for a wide variety of diseases such as asthma, rheumatism, allergies, and renal disorders, and their chronic use is associated with an increased risk of infectious diseases [29].

Another sub-group at possible higher risk would be travelers; according to surveillance data collected in the region of Waterloo, 19% of reported cases of campylobacteriosis were associated with international traveling [30]. Travel-associated cases are likely overestimated in surveillance databases, because people traveling abroad in the days preceding a diarrheal episode are more likely to seek medical advice, even after adjustment for disease severity [31]. It is possible that people do develop immunity against *Campylobacter*, but that such immunity is not effective when exposed to uncommon strains or to a highly contaminated environment when traveling in developing countries. In the Quebec surveillance database on reportable diseases, data on the occupation of the patient and whether cases are related to out of province travel were missing for 85% and 96% of patients respectively, making any analysis unfeasible. From another perspective, people with a previous diagnosis of campylobacteriosis might be more inclined to consult their physician when they experience another episode of acute gastroenteritis. In fact, the overall risk of campylobacteriosis in Canada has been estimated to be approximately 1.5% per year [2], which is very close to our 1.2% estimate.

The higher rate of recurrence for those with a previous infection of *Campylobacter *is suggestive of long-term or recurrent rather than punctual exposure, for example via an occupational exposure to animals, a source of drinking water or food habits [32-34]. This could be of particular importance for people living in rural areas where infection through drinking raw milk or being in direct contact with excreting farm animals is more likely [32,35]. This could be also true for young children who have closer contact with their environment due to specific behaviors. A long-term or recurrent exposure from a specific source is also suggested when *Campylobacter coli *is frequently isolated (i.e. 36%) in successive episodes but not commonly isolated in the general population. *Campylobacter coli *is known to be strongly associated with swine and certain environmental sources [36]. These speculations are supported by our results which show that the risk of campylobacteriosis following a first reported episode is higher in infants and people from rural regions.

Some assumptions underpinning this study must be mentioned. First, a proxy estimation was used for the date of campylobacteriosis onset, which is a potential source of error. However, we do not have reason to believe that this method biases the results. Moreover, the possibility that chronic cases were identified as recurrent cases is low. In fact, cases occurring less than 3 months from first episodes were excluded, and campylobacteriosis typically lasts less than 7 days [37,38] and bacterial excretion following the resolution of clinical signs persists for only two to nine weeks [7,39]. It was assumed that all people having had a reported episode of campylobacteriosis remained at risk of having another one up to the end of the study. However, information on patient mortality occurring after the episode was not available, and episodes reported in two different health regions for a single patient were not necessarily identified by a single identifier. Despite this fact, any death or move would have led to a reduction in the number of reported episodes, and ultimately to the same conclusions. Based on our observations and other reports, the fatality rate associated with reported campylobacteriosis episodes in Canada is typically less than 1% [40]. Likewise, the impossibility of linking cases occurring in a single patient but reported in different health regions would also lead to an underestimation of the risk of recurrent episodes, because this would lead to underestimating the number of cases of recurrent episodes. Finally, a misclassification bias could be present in our study, since patients with only one reported episode could have had another episode prior to our study time period. Likewise, not all patients stayed in the same region type for the entire study period; city dwellers can also move to cottages on a regular basis for short or long periods of time, yet remain classified as urban for statistical purposes. These misclassifications, if present, would have likely produced more conservative results with a reduction of the risk difference observed.

We used a logistic model to study the risk of recurrent episodes according to patient characteristics. This model did not take into account the correlation between individuals included twice in the analysis. Attempts were made to fit models using multi-level or generalized estimating equation methods but no convergence was obtained. The restriction of the analysis to patients having had a first episode would have led to misleading results due to the inability to adjust forr stratum-specific baseline risks. Development and validation of statistical models for studying recurrent episodes in this setting could be an issue to consider [41].

Data arising from surveillance systems, although imperfect, represent an invaluable source of information for studying the epidemiology of infectious diseases such as campylobacteriosis. However, given the experience of this study, such data would be further enhanced by capturing information related to domestic travel, underlying medical conditions, antacid or antibiotic use, and occupational information.

## Conclusions

When compared to a baseline risk, the risk for a recurrent episode of campylobacteriosis was increased for four years and followed a decreasing trend. This increased risk of a recurrent event was similar across gender, but higher for people from rural areas and lower for children under four years old. These results may suggest the absence of durable immunity or clinical resilience following a first episode of campylobacteriosis and periodic re-exposure, at least among cases reported through the surveillance system. A follow-up study on recurrent cases might be useful for understanding specific patient characteristics involved in this higher risk of recurrence. These results also suggest that recurrent cases of campylobacteriosis should be excluded from risk factor analyses, or that the correlation between episodes occurring in the same individual should be taken into account. Lastly, this data revealed some level of specificity in the epidemiology of campylobacteriosis for population sub-groups such as children and people living in rural areas. Patients experiencing campylobacteriosis would benefit from being informed about potential sources of contamination and adequate preventive measures.

## Competing interests

The authors declare that they have no competing interests.

## Authors' contributions

JA carried out data validation, data analysis, and drafted the manuscript. OB assisted with statistical analysis. All authors participated in study methodology and discussion. All authors read and approved the final manuscript.

## Pre-publication history

The pre-publication history for this paper can be accessed here:

http://www.biomedcentral.com/1471-2458/11/32/prepub

## Supplementary Material

Additional file 1 **Proxy dates for estimating date of onset**.Availability of proxy dates for estimating the date of onset and median interval between date of onset and proxy dates among reported cases of campylobacteriosis, by health regions, Quebec, 1996-2006 (Table). Click here for file
