# Supplementary material for: Do patients with recurrent episodes of campylobacteriosis differ from those with a single disease event?
Source: BMC Public Health. 2011 Jan 12;11:32. doi: 10.1186/1471-2458-11-32 (PMC3025843; doi:10.1186/1471-2458-11-32)
Supplement: Additional file 1 — Proxy dates for estimating date of onset.Availability of proxy dates for estimating the date of onset and median interval between date of onset and proxy dates among reported cases of campylobacteriosis, by health regions, Quebec, 1996-2006 (Table). [file 1471-2458-11-32-S1.PDF]

Additional file 1. Proxy dates for estimating date of onset

Availability of proxy dates for estimating the date of onset and median interval between date of onset and proxy dates among reported cases of campylobacteriosis, by health regions, Quebec, 1996-2006.

Health region	Number of cases	Physician declaration		Reception of physician declaration		Sample collection		Laboratory declaration		Reception of laboratory declaration	
		n	Median	n	Median	n	Median	n	Median	n	Median
1	943	44	9	54	12	407	4	514	7	519	10
2	1,569	435	13	412	17	109	4	968	9	972	11
3	4,237	34	7	35	11	187	4	215	7	220	9
4	2,473	211	11	217	16	912	4	1,060	8	1,080	11
5	1,489	98	14	102	21	879	4	910	9	927	12
6	5,300	416	14	427	20	577	3	615	8	714	12
7	896	169	8	170	16	129	0	386	6	423	9
8	333	72	12	72	16	56	3	240	13	240	15
9	307	13	7	13	9	9	0	51	6	51	8
11	304	38	12	36	17	18	4	57	8	60	12
12	2,216	68	9	84	12	674	4	1,212	7	1,221	10
13	1,013	19	10	20	24	14	5	44	6	45	16
14	1,444	346	11	357	15	1,090	4	1,134	8	1,160	10
15	1,948	37	9	36	14	64	5	800	4	846	11
16	4,989	1,066	12	1,104	21	2,496	0	2,553	5	2,611	11
Total	29,461	3,066	12	3,139	18	7,621	3	10,759	7	11,089	11
